# Supplementary figures and images for: Loss of lamin‐B1 and defective nuclear morphology are hallmarks of astrocyte senescence in vitro and in the aging human hippocampus
Source: Aging Cell. 2021 Dec 10;21(1):e13521. doi: 10.1111/acel.13521 (PMC8761005; doi:10.1111/acel.13521)

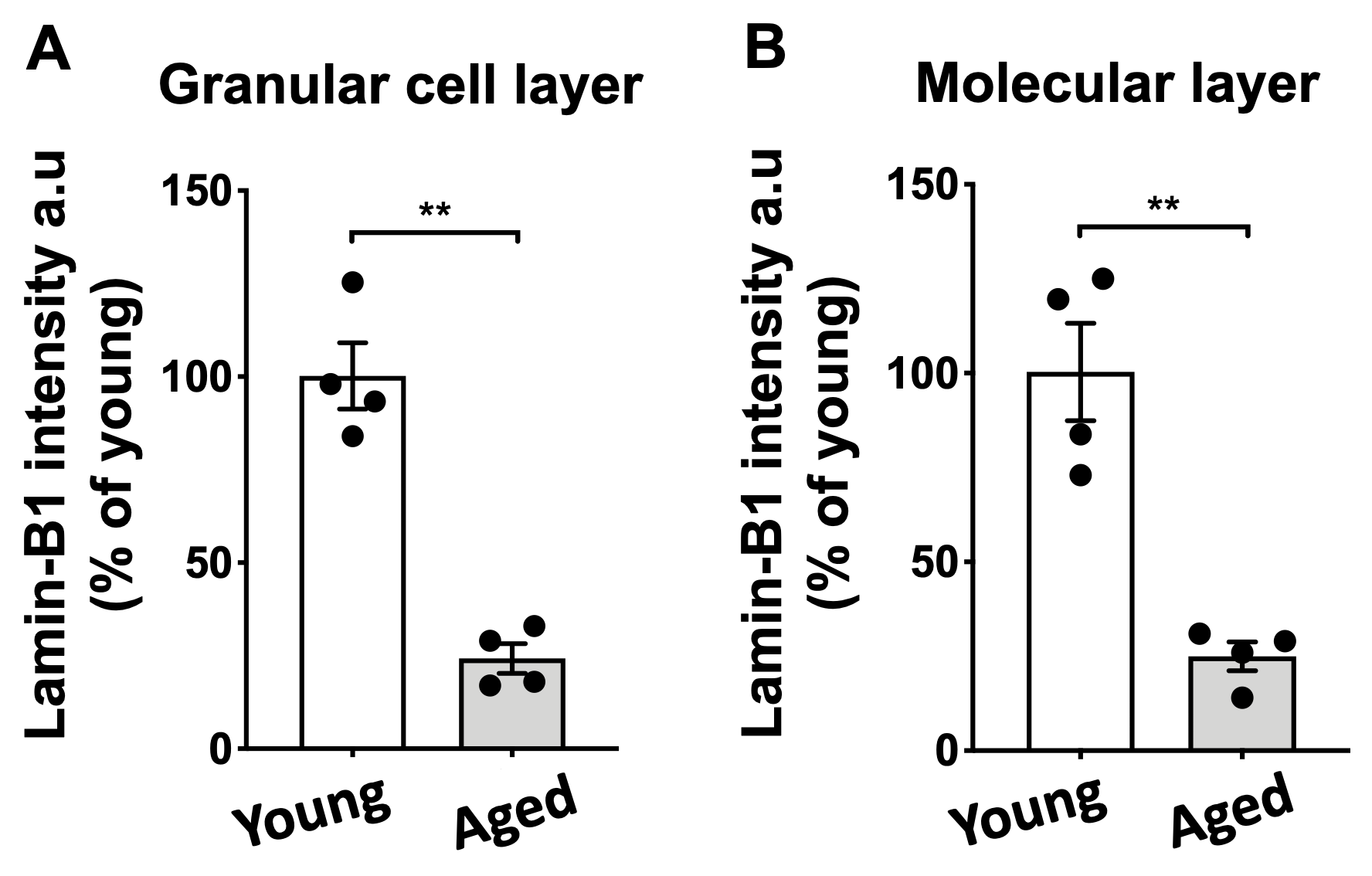

Supplement: Supplementary file 1 — Figure S1 [file ACEL-21-e13521-s001.tif]

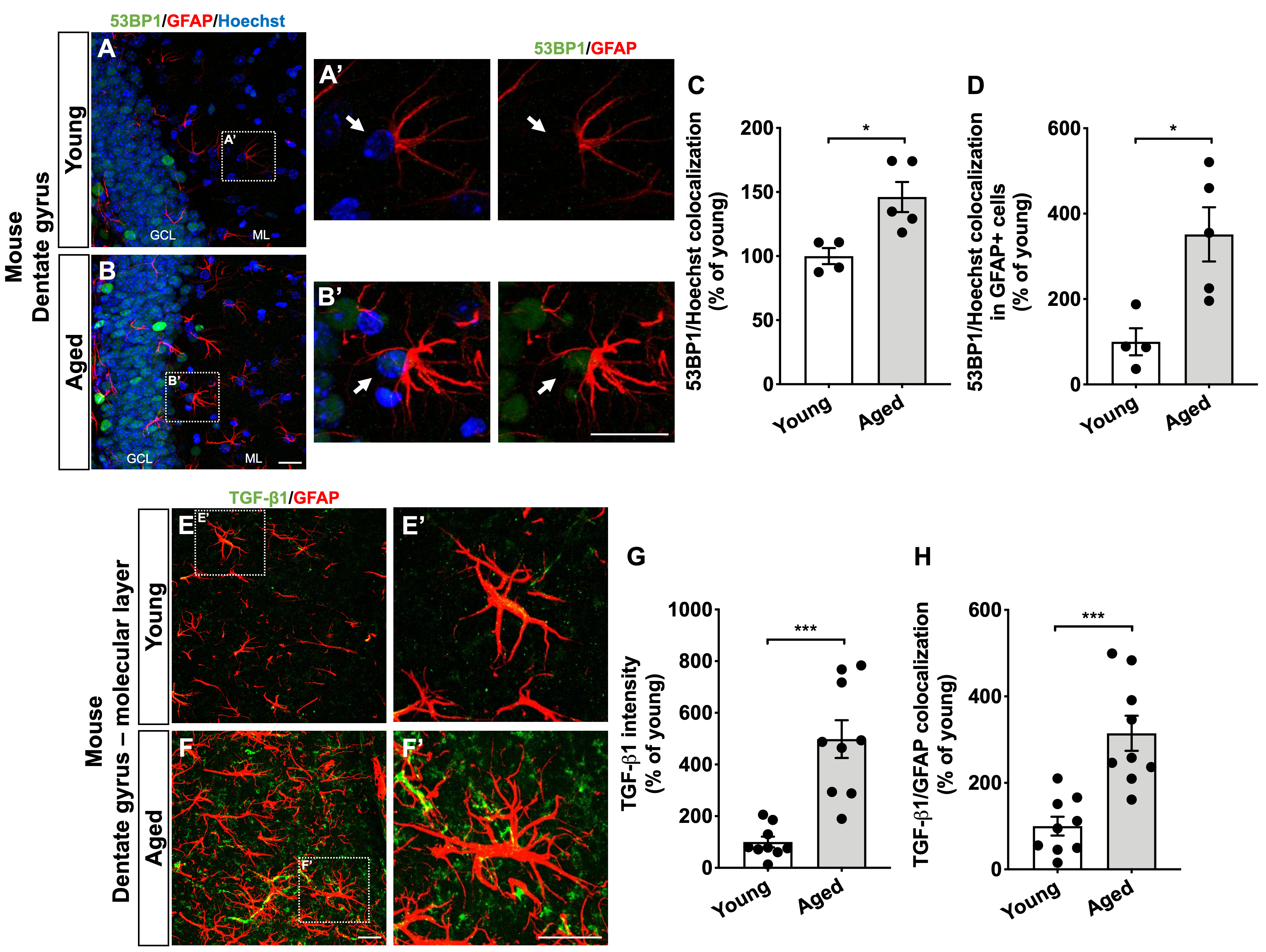

Supplement: Supplementary file 2 — Figure S2 [file ACEL-21-e13521-s004.tif]

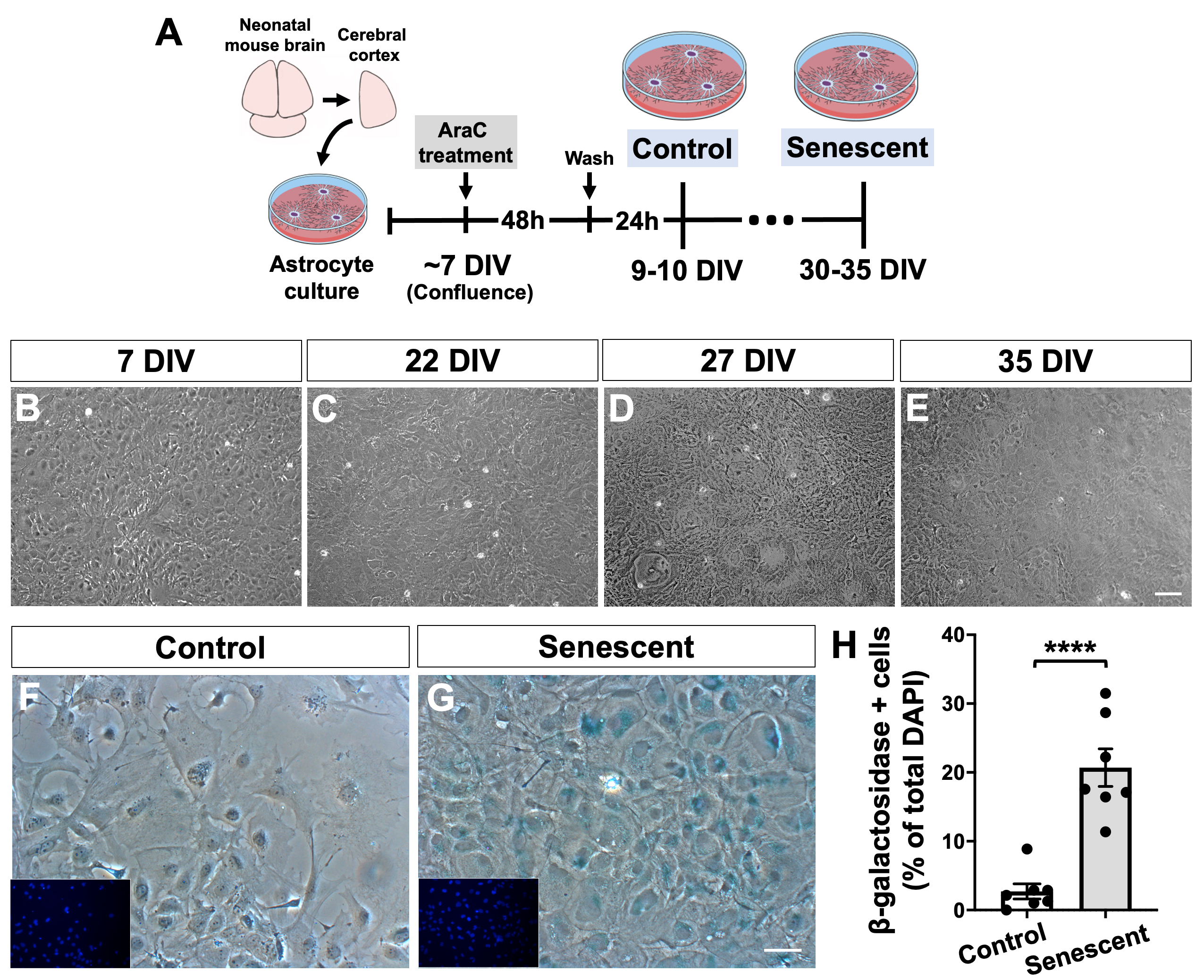

Supplement: Supplementary file 3 — Figure S3 [file ACEL-21-e13521-s002.tif]

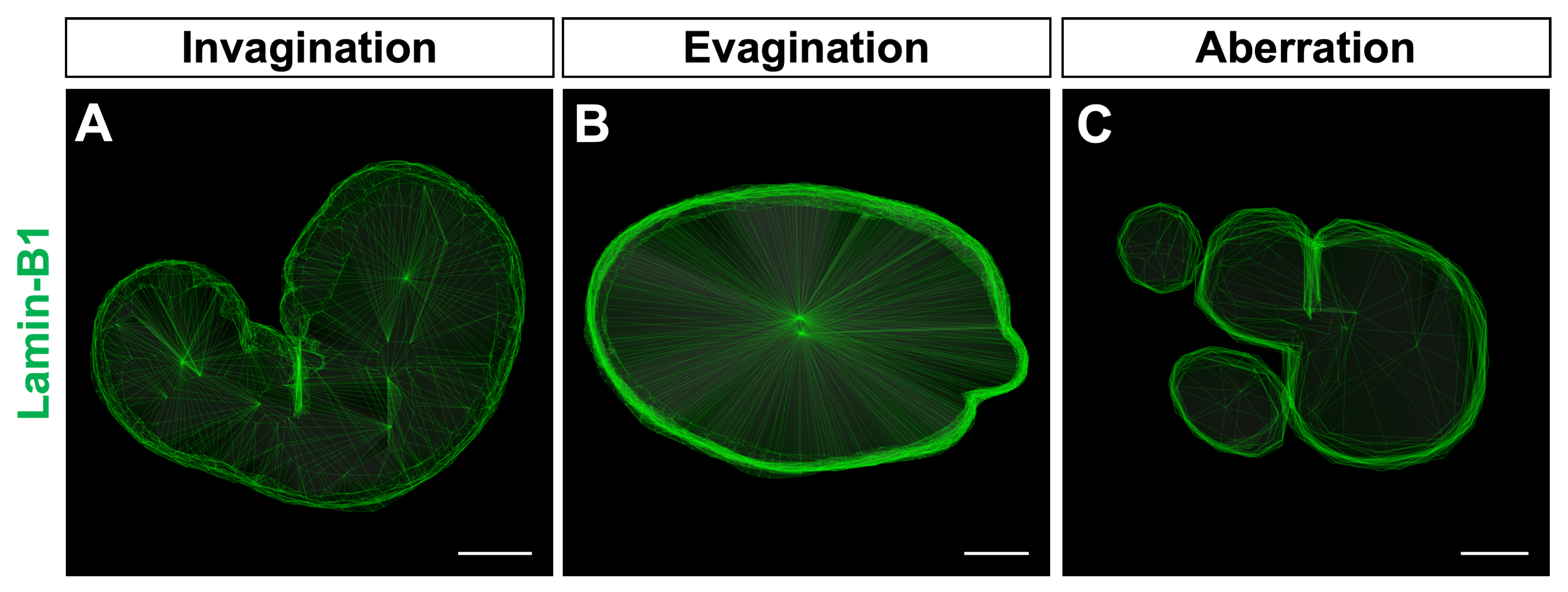

Supplement: Supplementary file 4 — Figure S4 [file ACEL-21-e13521-s008.tif]

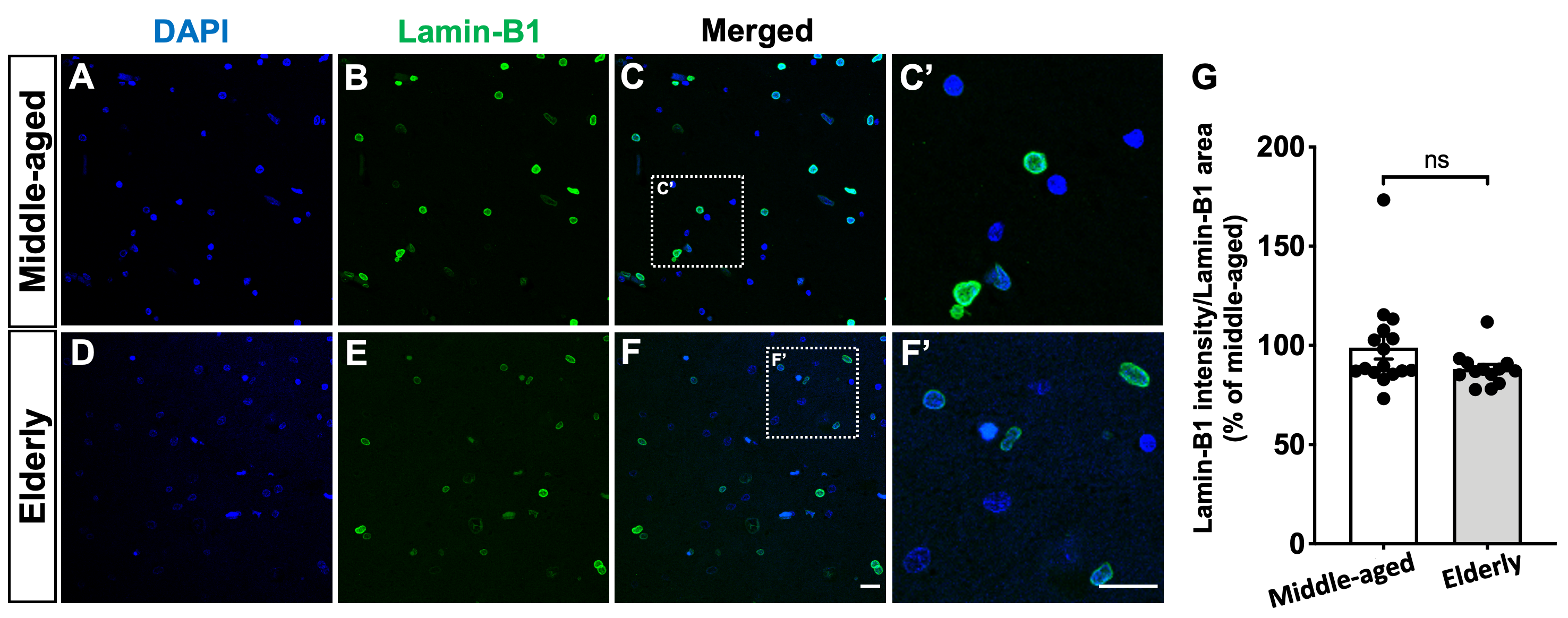

Supplement: Supplementary file 5 — Figure S5 [file ACEL-21-e13521-s009.tif]

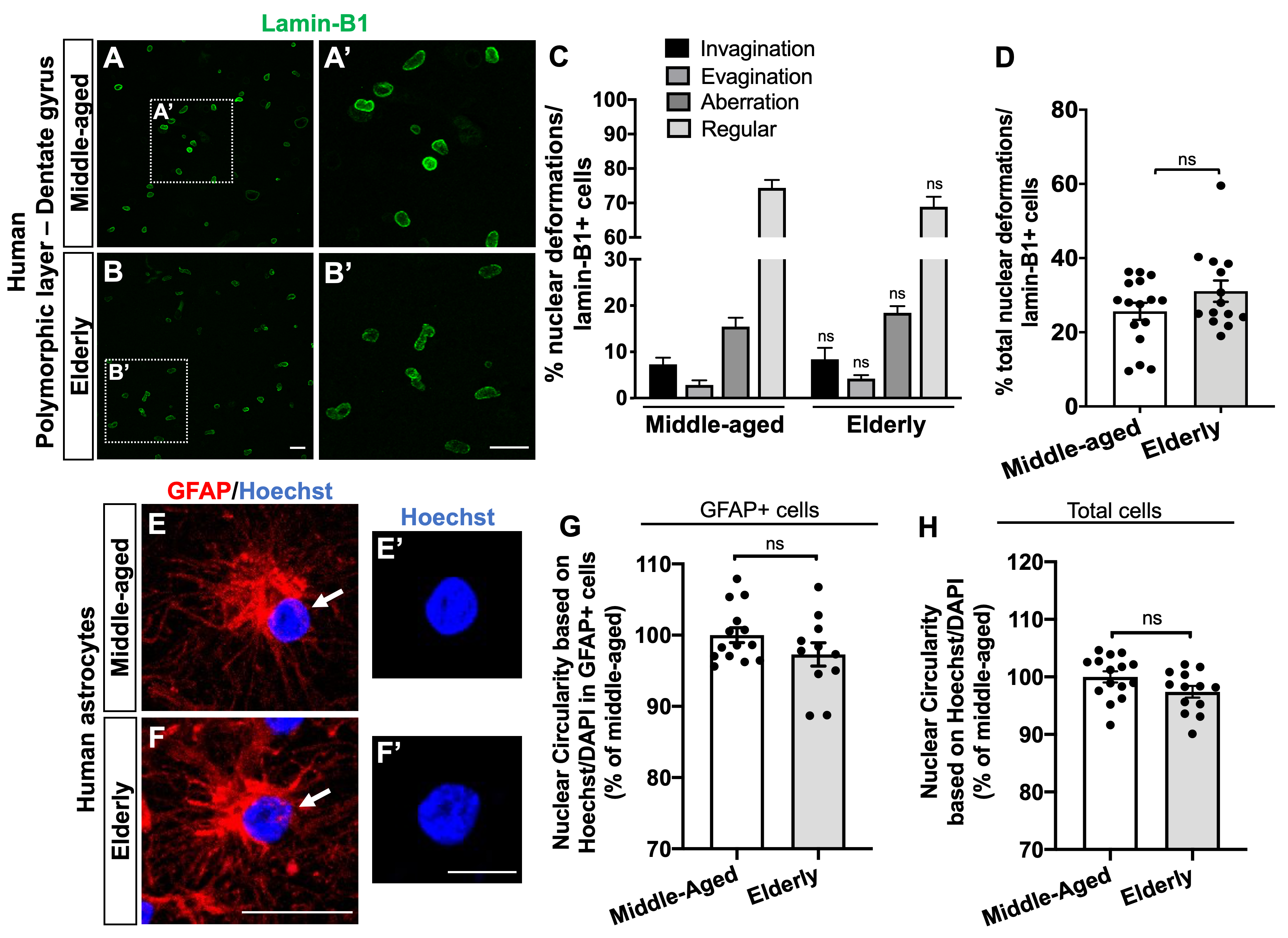

Supplement: Supplementary file 6 — Figure S6 [file ACEL-21-e13521-s007.tif]
