# Supplementary material for: Loss of lamin‐B1 and defective nuclear morphology are hallmarks of astrocyte senescence in vitro and in the aging human hippocampus
Source: Aging Cell. 2021 Dec 10;21(1):e13521. doi: 10.1111/acel.13521 (PMC8761005; doi:10.1111/acel.13521)
Supplement: Supplementary file 7 — Tables S1‐S2 [file ACEL-21-e13521-s003.pdf]

Matias et al., 2021
Supplementary Tables

Supplementary Table 1. Donors.

Sample	Sex	Age	Diagnosis	Amyloid	Braak	ApoE	PMD	pH CSF	Cause of death	Source
1991-118	m	59	Non-demented control	-	0	33	<06:00	6.05	Aspiration pneumonia and thrombo-emboli	NBB
1996-014	f	54	Non-demented control	0	0	33	08:00	6.45	Acute renal failure	NBB
1998-125	f	58	Non-demented control	-	1	43	06:15	6.31	Multiple organ failure	NBB
2005-034	m	56	Non-demented control	0	0	33	14:00	7.03	Terminal congestive heart failure	NBB
2011-081	m	55	Non-demented control	0	0	33	07:30	6.88	*Euthanasia with esophageal cancer	NBB
2012-071	f	57	Non-demented control	-	0	32	07:40	6.47	*Euthanasia with metastatic urothelial cancer	NBB
FLA24	f	60	Non-demented control	0	1	-	11:18	-	Pulmonary embolism	BBBABSG
FLA9	m	56	Non-demented control	0	2	34	18:00	6.00	Heart failure	BBBABSG
FLA3	f	56	Non-demented control	A	1	33	13:00	7.00	Cirrhosis	BBBABSG
FLA6	f	53	Non-demented control	-	-	-	21:42	6.80	Heart faliure	BBBABSG
FLA21	f	50	Non-demented control	0	I	-	10:36	-	Pneumonia	BBBABSG
FLA12	f	56	Non-demented control	0	0	-	16:24	-	Myocardial infarction	BBBABSG
FLA15	f	54	Non-demented control	0	2	33	09:12	-	Pulmonary edema	BBBABSG
FLA18	f	57	Non-demented control	0	2	-	11:18	-	Myocardial infarction	BBBABSG
FLA30	f	59	Non-demented control	-	-	-	15:06	7.00	Acute pancreatitis	BBBABSG
FLA27	m	60	Non-demented control	0	0	33	12:15	6.90	Heart failure	BBBABSG
2000-007	m	85	Non-demented control	0	2	33	15:10	6.85	Myocard infarction	NBB
2005-020	m	79	Non-demented control	A	1	33	06:30	6.32	Cerebrovascular accident	NBB
2005-061	f	93	Non-demented control	0	2	33	05:50	-	Cachexia (mamma carcinoma)	NBB
2006-049	f	84	Non-demented control	0	1	33	04:45	6.26	Heart failure, lung emphysema and dehydration	NBB
2008-054	f	92	Non-demented control	A	1	-	07:00	6.55	Acute death, probably pulmonary emboly	NBB
2011-028	f	81	Non-demented control	0	1	33	04:25	6.67	Intestinal ischemia	NBB
2011-111	m	93	Non-demented control	0	1	33	05:05	-	Heart failure	NBB

2013-016	m	83	Non-demented control	A	1	33	05:15	6.60	Myocardial infarction and palliative sedation	NBB
FLA11	f	76	Non-demented control	A	1	33	15:12	6.74	Heart failure	BBBABSG
FLA8	m	84	Non-demented control	0	2	23	17:48	6.74	Heart failure	BBBABSG
FLA5	f	79	Non-demented control	0	2	33	11:36	6.17	Pulmonary thromboembolism	BBBABSG
FLA14	f	79	Non-demented control	0	2	-	16:48	6.20	Pulmonary edema	BBBABSG
FLA20	f	77	Non-demented control	A	2	34	11:18	6.50	Heart failure	BBBABSG
FLA23	f	91	Non-demented control	A	2	33	17:00	-	Coronary artery disease	BBBABSG

NBB = Netherlands Brain Bank number; BBBABSG: Brain Bank of the Brazilian Aging Brain Study Group; m = male, f = female; ApoE = Apolipoprotein E; PMD = *Post-mortem* Delay in hours: min; CSF: Cerebrospinal fluid. *Euthanasia is legal in the Netherlands.

Supplementary Table 2. Number of nuclei analyzed for the nuclear deformation and circularity measurements in *post-mortem* human hippocampal tissue

**Marker**	**Measurement**	**Middle-aged**			**Elderly**		
		GCL	PL	Nº of donors	GCL	PL	Nº of donors
Lamin-B1+ nuclei	Nuclear deformation	4,191	776	16	4,522	900	14
Hoechst/DAPI nuclei	Nuclear circularity	741	522	15	715	524	13
Hoechst/DAPI of GFAP+ cells	Nuclear circularity	156	147	14	136	142	12
