# Supplementary material for: Loss of lamin‐B1 and defective nuclear morphology are hallmarks of astrocyte senescence in vitro and in the aging human hippocampus
Source: Aging Cell. 2021 Dec 10;21(1):e13521. doi: 10.1111/acel.13521 (PMC8761005; doi:10.1111/acel.13521)
Supplement: Supplementary file 11 [file ACEL-21-e13521-s010.docx]

**Matias et al., 2021**

**Supplementary Figures Legends**

**Supplementary Figure 1. Age-related decline of lamin-B1 in the mouse granular cell layer and molecular layer of the hippocampus.** Densitometric analysis of lamin-B1 staining in the granular cell layer (A) and molecular layer (B) revealed a significant reduction of lamin-B1 intensity in aged mice compared with young mice (p=0.0012 and p=0.0073, respectively). Significance was determined using Unpaired t test with Welch's correction. Error bars represent ± SEM. Individual data points are plotted and represent individual animals (*n*=4 animals per experimental group).

**Supplementary Figure 2. Senescence-related biomarkers are increased in aged mouse hippocampal astrocytes.** (A-B’) Immunostaining for 53BP1 and GFAP in the mouse hippocampal dentate gyrus of young and aged mice. (C, D) Aged mice showed a global increase in the colocalization of 53BP1 in Hoechst labeled nuclei (p=0.0135), including in GFAP positive cells, compared with young mice (p=0.0130). *n*= 4 young and 5 aged animals. (E, F’) Immunostaining for TGF-β1 and GFAP in the molecular layer of the dentate gyrus of young and aged mice. (G) Aged mice presented higher intensity of TGF-β1 (p=0.0005). (H) Colocalization ratio of TGF-β1 in GFAP+ cells increased in aged mice compared with young ones (p=0.0005). *n*= 9 animals per experimental group. Significance was determined using Unpaired t test with Welch's correction. Error bars represent ± SEM. Individual data points are plotted and represent individual animals. Scale bars, 20 µm.

**Supplementary Figure 3. Morphological view and percentage of β-galactosidase+ cells in control and senescent astrocyte cultures*.*** (A) To establish an *in vitro* model for astrocyte senescence, astrocyte cultures derived from murine cerebral cortex were treated by AraC for 48 h, washed, and then maintained for 9-10 DIV (control group) or 30-35 DIV (senescent group). (B-E) Astrocyte cultures were visualized at different time-points by phase contrast microscopy. Cultures showed a similar morphological profile after 35 DIV, characterized by a typical monolayer formed after 7 DIV (B) and an astrocytic flat-protoplasmic morphology. (F-H) Senescent astrocyte cultures showed an increased percentage of β-galactosidase+ cells compared with control cultures (p<0.0001). *n*=7 astrocyte cultures per experimental condition. Significance was determined using Unpaired t-test. Error bar represents ± SEM. Individual data points are plotted and represent individual cultures. Scale bars, 100 µm in (E) and 50 µm in (G).

**Supplementary Figure 4. Classification of nuclear deformations in astrocyte cultures.** Three-dimensional reconstruction of lamin-B1+ nuclei based on z-stack fluorescence microscopy was performed and three types of nuclear deformations were classified: (A) Invagination: invaginated nuclei showed one clear lamin-B1 invagination into the deep nuclear interior. (B) Evagination: evaginated nuclei exhibited one clear lamin-B1 protrusion from the nuclear lamina. (C) Aberration: aberrant nuclei showed a combination of more than one invagination, evagination or additional nuclear abnormalities. Scale bars, 5 µm.

**Supplementary Figure 5. Lamin-B1 intensity does not change in the polymorphic layer of the human hippocampus upon aging.** (A-G) Densitometric analysis revealed similar intensity of lamin-B1 staining at the polymorphic layer of *post-mortem* human tissue from middle-aged and elderly donors (p=0.0997). *n*=16 and 13 individuals for middle-aged and elderly groups, respectively. Scale bars, 20 µm in (F) and (F’). Significance was determined using Unpaired t test with Welch's correction. Error bars represent ± SEM. Individual data points are plotted and represent individual donors.

**Supplementary Figure 6. Neural cells at the polymorphic layer of the human hippocampus do not undergo age-dependent nuclear deformations.** (A-B) Distinct nuclear morphological profiles were evaluated, such as regular, evagination, invagination and aberration at the hippocampal polymorphic layer in *post-mortem* human tissue from middle-aged and elderly donors based on lamin-B1 staining. (C-D) Middle-aged and elderly donors exhibited a similar proportion of invaginated (p=0.6982), evaginated (p=0.2776) and aberrant nuclei (p=0.2311), as well as for the total nuclear deformations (i.e., evagination + invagination + aberration) (p=0.1510). *n* = 16 and 14 individuals for middle-aged and elderly groups, respectively. (E-F) Nuclear circularity was quantified based on Hoechst or DAPI staining at the hippocampal polymorphic layer in *post-mortem* human tissue from middle-aged (E-E’) and elderly donors (F-F’). (G) Astrocytes (GFAP+ cells) from both age groups showed similar nuclear circularities (p=0.1809). *n*=14 and 11 individuals for middle-aged and elderly groups, respectively. (H) Nuclear circularity of the total cells analyzed was also similar between the age groups (p=0.0759). *n*=15 and 13 individuals for middle-aged and elderly groups, respectively. Scale bars, 20 µm in (B), (B’) and (F); 10 µm in (F’). Significance was determined using Unpaired t test with Welch's correction. Error bars represent ± SEM. Individual data points are plotted and represent individual donors.
